# Slim Body Weight Is Highly Associated With Enhanced Lipoprotein Functionality, Higher HDL-C, and Large HDL Particle Size in Young Women

**DOI:** 10.3389/fendo.2018.00406

**Published:** 2018-07-19

**Authors:** Ki-Hoon Park, Dhananjay Yadav, Suk-Jeong Kim, Jae-Ryong Kim, Kyung-Hyun Cho

**Affiliations:** ^1^Department of Medical Biotechnology, Yeungnam University, Gyeongsan, South Korea; ^2^Research Institute of Protein Sensor, Yeungnam University, Gyeongsan, South Korea; ^3^LipoLab, Gyeongsan, South Korea; ^4^Department of Biochemistry and Molecular Biology, Smart-Aging Convergence Research Center, College of Medicine, Yeungnam University, Daegu, South Korea

**Keywords:** body weight, blood pressure, lipoproteins, apoA-I, HDL-cholesterol

## Abstract

There has been no information about the correlations between body weight distribution and lipoprotein metabolism in terms of high-density lipoproteins-cholesterol (HDL-C) and cholesteryl ester transfer protein (CETP). In this study, we analyzed the quantity and quality of HDL correlations in young women (21.5 ± 1.2-years-old) with a slim (*n* = 21, 46.2 ± 3.8 kg) or plump (*n* = 30, 54.6 ± 4.4 kg) body weight. Body weight was inversely correlated with the percentage of HDL-C in total cholesterol (TC). The plump group showed 40% higher body fat (26 ± 3 %) and 86% more visceral fat mass (VFM, 1.3 ± 0.3 kg) than the slim group, which showed 18 ± 2% body fat and 0.7 ± 0.2 kg of VFM. Additionally, the plump group showed 20% higher TC, 58% higher triglyceride (TG), and 12% lower HDL-C levels in serum. The slim group showed 34% higher apoA-I but 15% lower CETP content in serum compared to the plump group. The slim group showed a 13% increase in particle size and 1.9-fold increase in particle number with enhanced cholesterol efflux activity. Although the plump group was within a normal body mass index (BMI) range, its lipid profile and lipoprotein properties were distinctly different from those of the slim group in terms of CETP mass and activity, HDL functionality, and HDL particle size.

## Introduction

Maintenance of a slim body shape is greatly desired for health and beauty purposes, especially in young women. However, there has been no information about the correlations between body weight distribution and lipoprotein metabolism. It is well known that serum high-density lipoprotein-cholesterol (HDL-C) level is inversely correlated with the incidence of coronary artery disease (1). HDL plays important roles in promoting anti-atherosclerotic, anti-diabetic, and anti-thrombotic activities (2) in serum and interacts with many antioxidant enzymes such as paraoxonase (3, 4). However, HDL can be altered under various health conditions (5), such as changed dietary patterns (6), pathogen infection (7), and environmental stress (8). Therefore, HDL-C may be a good biomarker for the diagnosis of many diseases and disease progression by monitoring changes in its antioxidant and anti-inflammation abilities (9). However, it is well known that dysfunctional HDL is frequently found in patients with metabolic diseases such as obesity (10).

Although overweight status and obesity are associated with low HDL-C levels, there has been no report characterizing HDL particles from overweight subject. In the context of metabolic syndrome, obese persons (body mass index, BMI >30) with insulin resistance were shown to have low HDL-C levels, specifically 41% in women (<45 mg/dL), and 31% in men (<35 mg/dL) (11). Reduction of the HDL-C level is linked with increased very low-density lipoprotein (VLDL) production via elevation of cholesteryl ester transfer protein (CETP) activity. Further, obese subjects often show increased CETP activity (12, 13).

Apolipoprotein A-I (apoA-I), the major protein of HDL, exerts antioxidant and anti-inflammatory activities in lipid-free and lipid-bound states along with cholesterol efflux activity (14) in macrophages and insulin secretion activity in pancreatic beta cells. During the progression of obesity or insulin resistance in early metabolic syndrome, tumor necrosis factor-α (TNF-α) is up-regulated while apoA-I production is down-regulated. On the other hand, apoA-I gene expression can be down-regulated by increased production of adipokines and inflammatory cytokines (15, 16).

Progression of overweight status is associated with various complications such as hypertension, hyperglycemia, and coronary artery disease mediated via several mechanisms, including reduced HDL-C and elevated total cholesterol (TC), low-density lipoprotein cholesterol (LDL-C), and triglyceride (TG) levels (17). Although a high quantity of serum lipids has been well established as a cause of obesity and hypertension in adolescents, there has been no report on the quality of lipids and lipoproteins according to body weight. Furthermore, there has been no report comparing lipoprotein quality between two groups (slim and plump) showing a slight difference in body weight within a normal range, whereas lipid and lipoprotein properties of obese patients have been relatively well investigated.

In the current study, we compared lipoprotein biomarkers between slim (around 46 kg) and plump (around 55 kg) young women groups, although both are considered as having a normal body weight. Between the plump and slim type, lipid and lipoprotein qualities were compared to identify new biomarkers contributing to a slim body shape in young women.

## Materials and methods

### Subjects

We randomly recruited healthy female volunteers (*n* = 51) enrolled at Yeungnam University in 2015. Heavy alcohol consumers (>30 g EtOH/day) and those who had consumed any prescribed drugs to treat hyperlipidemia, diabetes mellitus, or hypertension were excluded. All subjects had unremarkable medical records without illicit drug use or past history of systemic diseases. The study was approved by the Institutional Review Board at Yeungnam University (Gyeongsan, South Korea) endorsed the protocol (IRB #7002016-A-2016-021) and the participants signed an informed consent form prior to research commencement.

### Anthropometric analysis

Blood pressure was measured each morning at 2-week intervals by an Omron HEM-1000 (Kyoto, Japan). Height, body weight, BMI, total body fat (%), total body fat mass (kg), and visceral fat mass (VFM) (kg) were measured individually at the same time of day at 2-week intervals using an X-scan plus II body composition analyzer (Jawon Medical, Gyeongsan, Korea).

### Vascular stiffness analysis

Systolic and diastolic blood pressures were measured in triplicate using a semi-automated non-invasive oscillometric sphygmomanometer using the SphygmoCor system (Actor Medical, Sydney, Australia) following a 5 min rest period (18). Pulse wave was analyzed with SphygmoCor software. Augmentation pressure is the gain in aortic pressure above its first systolic push, which is thought to be determined by pressure wave reflection but may be influenced by ventricular ejection aspects. However, the augmentation index, the ratio of augmentation pressure to aortic or central pulse pressure, is widely used as a measure of pressure wave reflection (19, 20). Measurements were performed by a technician trained in the technique and blinded to the characteristics of each subject.

### Plasma analysis

Blood was obtained from all subjects following overnight fasting. Blood was collected using a vacutainer (BD Biosciences, Franklin Lakes, NJ, USA) containing EDTA (final concentration, 1 mM). Plasma was isolated by low-speed centrifugation (3,000 rpm) and stored at −80°C until analysis. To analyze plasma, TC, TG, HDL-C, glucose, aspartate aminotransferase (AST), and alanine aminotransferase (ALT) levels were measured using commercially available kits (Cleantech TS-S; Wako Pure Chemical, Osaka, Japan).

### Characterization of lipoproteins

Very low-density lipoprotein (VLDL, d <1.019 g/mL), LDL (1.019 <d <1.063), HDL_2_ (1.063 < d <1.125), and HDL_3_ (1.125 < d <1.225) were isolated from pooled plasma of each group via sequential ultracentrifugation (21), and the density was adjusted by addition of NaCl and NaBr in accordance with standard protocols. Samples were centrifuged for 22 h at 10°C at 100,000 g using a Himac CP-100NX (Hitachi, Tokyo, Japan) using P50AT4-0124 rotor in our laboratory. Protein concentrations of lipoproteins were determined via Lowry protein assay, as modified by Markwell et al. (22). Expression levels of apoA-I (28 kDa) and apo-B (550 kDa) were determined by SDS-PAGE.

To assess the degree of lipoprotein oxidation, the concentration of oxidized species in lipoproteins was determined by the thiobarbituric acid-reacting substance (TBARS) assay method using malondialdehyde as a standard (23). To compare the extent of glycation between groups, advanced glycation end products (AGEs) in lipoproteins were determined from reading fluorometric intensities at 370 nm (excitation) and 440 nm (emission), as our previous report (24), using a spectrofluorometer LS55 (Perkin Elmer, Shelton, CT, USA) with the WinLab software package (version 4.0).

### Cholesteryl ester transfer protein assay

A rHDL-containing apoA-I and cholesteryl oleate were synthesized in accordance with the method described by Cho (25) and Cho et al. (26) using trace amounts of [^3^H]-cholesteryl oleate (TRK886, 3.5 μCi/mg of apoA-I; GE Healthcare). The rHDL was immobilized using CNBr-activated Sepharose 4B resin (Amersham Biosciences) for easy separation after the reaction in accordance with the manufacturer's instructions. CE transfer reaction was performed in 300-μL reaction mixtures containing human serum (20 μL) or HDL_3_ (20 μL, 2 mg/mL) as a CETP source, [^3^H]-rHDL-agarose (20 μL, 0.25 mg/mL) as a CE-donor, and human LDL (20 μL, 0.25 mg/mL) as a CE-acceptor. After incubation of 4 h at 37°C, the reaction was halted via brief centrifugation (10,000 g) for 3 min at 4°C. The supernatant containing CE-acceptor (150 μL) was then subjected to scintillation counting, and percentage transfer of [^3^H]-CE from [^3^H]-rHDL to LDL was calculated.

### Ferric reducing ability of plasma assay and paraoxonase activity

The ferric reducing ability of plasma (FRAP) was determined using the method described by Benzie and Strain (27). The antioxidant activities of individual HDL fractions were estimated by measuring increases in absorbance produced by generated ferrous ions. Paraoxonase-1 (PON-1) activity was then determined by measuring the initial velocity of *p*-nitrophenol production at 37°C, as determined by measuring the absorbance at 405 nm (microplate reader, Bio-Rad model 680; Bio-Rad, Hercules, CA, USA), as described previously (28) with slight modifications (29). Prior to the measurement, HDL was extensively dialyzed against PBS to remove EDTA.

### Phagocytosis of LDL into macrophages

THP-1 cells, a human monocytic cell line, were obtained from the American Type Culture Collection (ATCC, #TIB-202^TM^, Manassas, VA, USA) and maintained in RPMI-1640 medium (Hyclone, Logan, UT) supplemented with 10% fetal bovine serum until needed. Cells that had undergone no more than 20 passages were incubated in medium containing phorbol 12-myristate 13-acetate (PMA, 150 nM) in 24-well plates for 48 h at 37°C in a humidified incubator (5% CO_2_, 95% air) in order to induce differentiation into macrophages. Differentiated and adherent macrophages were then rinsed with warm PBS, followed by incubation with 450 μL of fresh RPMI-1640 medium containing 0.1% FBS and 50 μg of each LDL (1 mg of protein/mL in PBS) for 48 h at 37°C in a humidified incubator. After incubation, cells were washed with PBS three times and then fixed in 4% paraformaldehyde for 10 min. Next, fixed cells were stained with oil-red O staining solution (0.67%) and washed with distilled water. THP-1 macrophage-derived foam cells were then observed and photographed using a Nikon Eclipse TE2000 microscope (Tokyo, Japan) at 400× magnification. Cell media (0.2 mL) were then analyzed by TBARS assay to evaluate changes in levels of oxidized species using malondialdehyde (MDA) as a standard.

### Anti-atherogenic activity of HDL_3_

Differentiated and adherent macrophages were then rinsed with warm PBS and incubated with 400 μL of fresh RPMI-1640 medium containing 0.1% fetal bovine serum, 50 μg of oxLDL (1 mg of protein/mL in PBS), and 30 μg of HDL_3_ (2 mg of protein/mL in PBS) from each group for 48 h at 37°C in a humidified incubator. After incubation, cells were stained with oil-red O solution (0.67%) to visualize the amount of lipid species in cells. THP-1 macrophage-derived foam cells were then observed and photographed using a Nikon Eclipse TE2000 microscope (Tokyo, Japan) at 400× magnification. The stained area was quantified via computer-assisted morphometry using Image Proplus software (version 4.5.1.22, Media Cybernetics, Bethesda, MD).

### Cholesterol efflux

THP-1 cells were incubated in medium containing phorbol 12-myristate 13-acetate (PMA, 150 nM) in a plate for 48 h at 37°C in a humidified incubator in order to induce differentiation into macrophages. Macrophages were treated with radiolabeled cholesterol (0.1 μCi of [^3^H]-cholesterol) in RPMI 1640 medium (Hyclone, Logan, UT) containing 1% fetal bovine serum (Hyclone, Logan, UT) per well (0.5 mL) for 48 h. Media containing the isotope was saved and replaced with fresh media containing 0.3 mM 8-(4-chlorophenylthio)-cyclic adenosine monophosphate (cAMP, Cat# C3912, Sigma-Aldrich, St. Louis, MO) to up-regulate the cellular cholesterol pump (ATP-binding cassette transporter 1, ABCA1) for 18 h. After removal of media containing cAMP, 28 μg of HDL_3_ was added and the sample incubated with serum-free media (0.5 mL) for 24 h. Subsequently, cell media (0.5 mL) in individual wells were collected into a 1.7 mL tube. Cells were then rinsed with PBS three times and dissolved in 0.2 mL of RIPA buffer (50 mM Tris-HCl [pH 8.0], 150 mM NaCl, 5 mM EDTA [pH 8.0], 1% NP-40, 0.5% sodium deoxycholate, 0.1% sodium dodecyl sulfate) to lyse cells. An aliquot of the cell lysate (0.1 mL) was mixed with a scintillation cocktail (3 mL) to quantify the amount of isotope indicating uptake of cholesterol into cells. After scintillation counting of [^3^H]-cholesterol in cells and media, the amount of effluxed cholesterol from cells was calculated using the following formula as previously reported (30):

$$\begin{array}{l} \%\text{ Cholesterol efflux }=\frac{\left(\text{media counts }\times \text{ dilution factor}\right)}{\left(\text{media count }\times \text{ dilution factor}\right)\;+\;\left(\text{cell lysis count }\times \text{ dilution factor}\right)}\;\times \;100\\ \%\text{ Net efflux }=\;\%\text{ cholesterol efflux }\left(\text{with HDL3}\right)\;-\;\%\text{ blank efflux }\left(\text{without HDL3}\right) \end{array}$$

### ELISA and western blotting

The quantity of apoA-I in serum was determined in each group by ELISA using a commercially available kit (Quantikine ELISA, DAPA10, R&D systems, Minneapolis, MN). In order to quantify serum CETP, each well of a polystyrene microplate (#3590; Corning Inc., Corning, NY, USA) was coated with anti-human CETP rabbit antibody (ab19012; Abcam, Cambridge, UK) at a concentration of 0.25 μg/mL, followed by incubation overnight at 4°C. Equally, diluted serum sample was incubated for 2 h at room temperature. After extensive washing, anti-human CETP mouse antibody (ab2726; Abcam, 1 μg/mL) was added and the sample incubated for 2 h at room temperature. To develop the color reaction, anti-mouse IgG antibody (ab6728; Abcam, 0.5 μg/mL) conjugated with horseradish peroxidase was added. For color development, 3,3′,5,5′-tetramethylbenzidine (TMB) substrate solution (Cat. No. 555214; BD Biosciences, Flanklin Lakes, NJ, USA) was added and quantified using a Victor X4 microplate reader (Perkin Elmer, Waltham, MA).

Apolipoprotein/lipoprotein compositions were compared via sodium dodecyl sulfate-polyacrylamide gel electrophoresis (SDS-PAGE) using identical protein loading quantities (3 μg of total protein per lane) from individual HDL_3_, and expression levels of apolipoprotein were analyzed via immunodetection. Anti-human apoA-I antibody (ab7613), anti-paraoxonase antibody (ab24261), and anti-IL-6 antibody (ab6672) were purchased from Abcam (Cambridge, UK). Relative band intensity (BI) was compared via band scanning with Chemi-Doc® XRS + (Bio-Rad, Hercules, CA) using Image Lab software (Version 5.2).

### Electron microscopy

Transmitted electron microscopy (TEM) was performed with a Hitachi electron microscope (model H-7600; Ibaraki, Japan) operated at 80 kV as in our previous report (31). HDL_2_ were negatively stained with 1% sodium phosphotungstate (pH 7.4) with a final apolipoprotein concentration of 0.3 mg/mL in TBS.

### Zebrafish and embryos

Wildtype zebrafish and embryos were maintained according to standard protocols. Zebrafish maintenance and experimental procedures were approved by the Committee of Animal Care and Use of Yeungnam University (Gyeongsan, Korea). Zebrafish and embryos were maintained in a system cage (3 L volume, acrylic tank) and 6-well plates, respectively, at 28°C during treatment under a 14:10 h light:dark cycle.

### Microinjection of zebrafish embryos

To compare antioxidant and anti-inflammatory activities of HDL between slim and plump, HDL_3_ from each group was injected into zebrafish embryos, as in our previous report (32). Embryos at 1-day post-fertilization (dpf) were individually microinjected using a pneumatic picopump (PV820; World Precision Instruments, Sarasota, FL, USA) equipped with a magnetic manipulator (MM33; Kantec, Bensenville, IL, USA) and a pulled microcapillary pipette-using device (PC-10; Narishigen, Tokyo, Japan). To minimize bias, injections were performed at the same position on each yolk. Filter-sterilized solution of each LDL or HDL_3_ was injected into flasks of embryos. Following injection, live embryos were observed under a stereomicroscope (Motic SMZ 168; Hong Kong) and photographed using a Motic cam2300 CCD camera.

### Imaging of reactive oxygen species (ROS)

Injection of HDL_3_ from slim and plump group into the zebrafish embryo in the presence of ox LDL was done and changes in reactive oxygen species (ROS) levels in larvae were imaged by dihydroethidium (DHE; cat # 37291, BioChemika) staining, as previously described (33). Images were obtained by fluorescence microscopy (Ex = 588 nm and Em = 605 nm) on a Nikon Eclipse TE2000 instrument (Tokyo, Japan). To avoid bias, red fluorescence was measured in the trunk area away from the injection site. Quantification of the stained area was carried out via computer-assisted morphometry using Image Proplus software (version 4.5.1.22, Media Cybernetics, Bethesda, MD).

### Data analysis

All data are expressed as the mean ± SD from at least three independent experiments with duplicate samples. Data comparisons were assessed by Student's *t*-test using the SPSS program (version 14.0; SPSS, Inc., Chicago, IL, USA). In the human study, data in the same group were evaluated via one-way analysis of variance (ANOVA) using SPSS (version 14.0; Chicago, IL, USA), and differences between the means were assessed using Duncan's multiple-range test. Statistical significance was defined as *p* < 0.05.

## Results

### BMI and serum HDL-C

All participants were very similar in age (21.5 ±1.2 years old) and height (161.5 ± 4.2 cm). Participants were of normal body weight and BMI but were divided into a plump group (around 55 kg of BW) and slim group (around 46 kg of BW).

The plump group showed a significantly higher BMI, body fat percentage, and VFM than the slim group, as shown in Table 1. The systolic blood pressure and diastolic blood pressure were not found to be significant between the slim and plump group.

**Table 1:** Anthropometric data.

	**Total (*n* = 51)**	**Plump (*n* = 30)**	**Slim (*n* = 21)**	***p-*value**
Age (years)	21.5 ± 1.2	21.9 ± 1.7	21.1 ± 0.5	0.0712
Height (cm)	161.5 ± 4.2	161.5 ± 4.3	161.6 ± 4.4	0.4728
Weight (kg)	50.2 ± 5.8	54.6 ± 4.4	46.2 ± 3.8^***^	<0.001
BMI (kg/m^2^)	19.2 ± 2.0	20.9 ± 1.3	17.7 ± 1.0^***^	<0.001
Body fat (%)	22.1 ± 4.4	25.9 ± 3.0	18.7 ± 2.1^***^	<0.001
Visceral fat mass (kg)	1.0 ± 0.4	1.3 ± 0.3	0.7 ± 0.2^***^	<0.001
Subcutaneous fat mass (kg)	10.3 ± 3.0	12.9 ± 2.1	8.0 ± 1.5^***^	<0.001
Systolic blood pressure (mmHg)	114.2 ± 9.6	117.0 ± 8.2	111.6 ± 10.3	0.1037
Diastolic blood pressure (mmHg)	68.5 ± 8.1	71.0 ± 8.6	66.2 ± 7.2	0.0896
Blood pulse (beats/min)	83.9 ± 10.3	82.8 ± 11.7	84.9 ± 9.3	0.3255
Augmentation Pressure (mmHg)	0.9 ± 2.7	3.0 ± 2.5	−0.2 ± 2.2	0.0137
Augmentation Index	3.3 ± 9.0	10.2 ± 5.6	−0.6 ± 8.4	0.0128
Reference age (years)	24.1 ± 9.1	30.0 ± 13.9	20.8 ± 2.3	0.0339

*** *p < 0.001 vs. plump group*.

### Serum lipid level and body shape

The plump group showed 1.2- and 1.6-fold higher serum TC and TG levels, respectively, than the slim group, although all TC and TG levels were within their normal ranges (Table 2). However, the plump group showed 12% lower HDL-C and 1.7-fold higher TG/HDL-C than the slim group. The slim group showed 1.3-fold higher % HDL-C and serum apoA-I content than the plump group. Serum uric acid level of the plump group was slightly higher than that of the slim group. However, there was no significant difference in serum glucose, uric acid, or AST/ALT level between the groups.

**Table 2:** Serum profile.

	**Total (*n* = 31)**	**Plump (*n* = 20)**	**Slim (*n* = 11)**	***p*-value**
TC (mg/dL)	188.3 ± 35.9	206.1 ± 31.1	172.1 ± 33.1^*^	0.0129
TG(mg/dL)	63.3 ± 24.6	78.4 ± 27.3	49.7 ± 10.9^**^	0.0035
HDL-C (mg/dL)	70.5 ± 11.9	65.9 ± 8.2	74.7 ± 13.6^*^	0.0454
%HDL-C	38.4 ± 8.6	32.6 ± 6.2	43.8 ± 6.9^***^	0.0005
TG/HDL-C	0.9 ± 0.4	1.2 ± 0.5	0.7 ± 0.2^**^	0.0032
LDL-C (mg/dL)	106.8 ± 33.8	126.0 ± 32.4	89.5 ± 25.5^**^	0.0070
%LDL-C	54.7 ± 8.4	59.3 ± 7.4	50.5 ± 7.2^**^	0.0086
apoA-I (mg/mL)	2.7 ± 1.2	2.3 ± 1.1	3.1 ± 1.2^*^	0.0467
CETP mass	2.1 ± 0.3	2.3 ± 0.3	2.0 ± 0.3^*^	0.0477
%CE-transfer	28.9 ± 3.4	30.6 ± 3.9	27.4 ± 1.9^*^	0.0202
Glucose (mg/dL)	79.5 ± 5.7	80.5 ± 5.1	78.6 ± 6.2	0.2300
Uric acid (mg/dL)	5.8 ± 1.1	6.1 ± 1.1	5.6 ± 1.0	0.1380
AST (karmen/mL)	15.8 ± 1.5	15.5 ± 1.4	16.1 ± 1.6	0.1838
ALT (karmen/mL)	15.5 ± 2.2	14.9 ± 2.0	16.1 ± 2.3	0.1087

* p < 0.05;

** p < 0.01;

*** *p < 0.001 vs. plump group*.

### CETP mass and activity

Interestingly, the plump group showed significantly higher CETP mass than the slim group (Table 2). Furthermore, there was a significant difference in serum CETP activity (*p* = 0.0202) between the slim and plump groups although around 10% of activity was different (Table 2).

### Correlation of HDL-C and body shape

**Figure 1:**
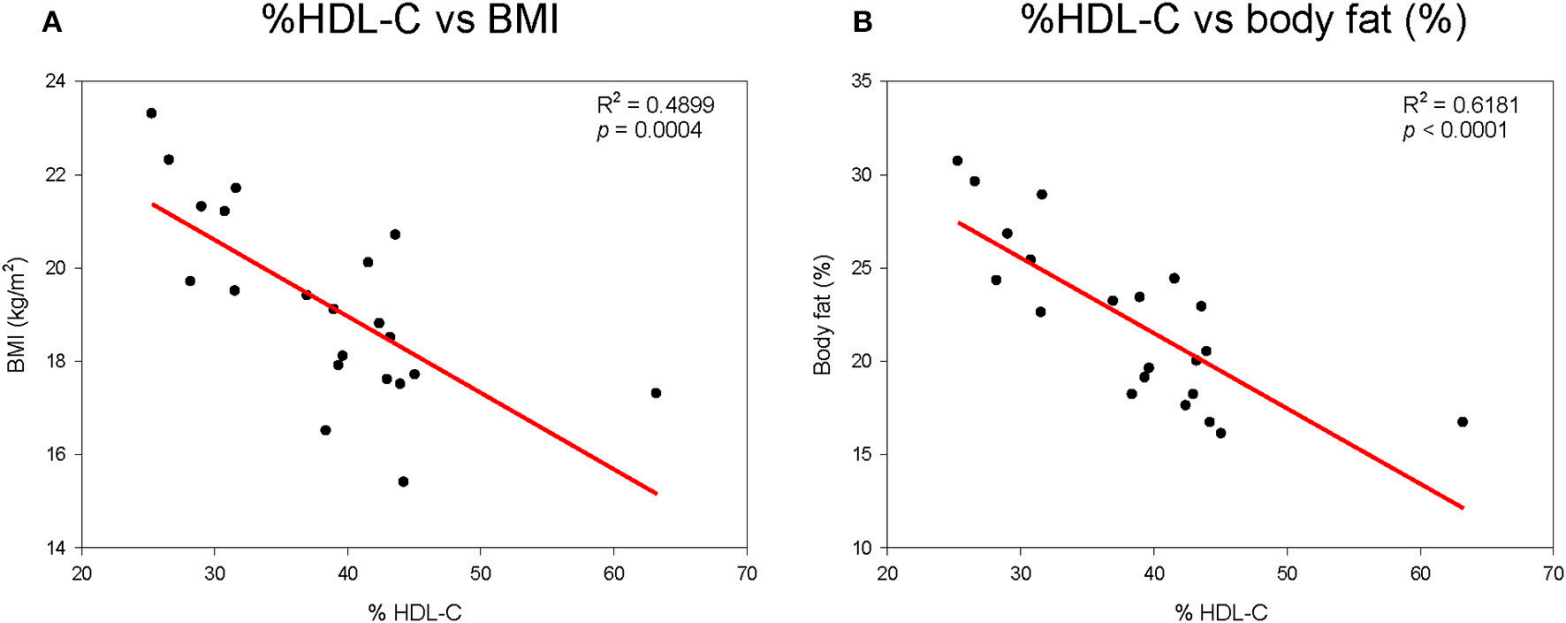
Correlation of % HDL-C with body mass index **(A)** and body fat mass **(B)**.

As shown in Supplementary Figure 1A, height generally increased with elevation of % HDL-C. Although there was no statistical difference, height and % HDL-C showed positive correlations. However, % HDL-C was inversely correlated with body weight, as shown in Supplementary Figure 1B. Correlations between BMI and % HDL-C (Figure 1A) and between % HDL-C and % body fat mass (Figure 1B) also showed clear inverse relationships. Visceral fat mass was inversely correlated with % HDL-C (*p* < 0.0001, Supplementary Figure 2A). However, visceral fat was positively correlated with CETP mass (Supplementary Figure 2B).

### Correlation of HDL-C and blood pressure

Both systolic and diastolic blood pressures were negatively correlated with % HDL-C as shown in Supplementary Figure 3. Diastolic blood pressure and % HDL-C showed steeper slopes than systolic blood pressure, suggesting that % HDL-C was more closely associated with lowered diastolic blood pressure (*p* = 0.0031). The plump group showed 5–6 mmHg higher diastolic and systolic blood pressures than the slim group.

The plump group showed a remarkably high central augmentation pressure and index with an arterial stiffness reference age of 30 ± 13.9-years-old, whereas the slim group showed a reference age of 20.8 ± 2.3-years-old (Table 1).

### Expression level of apoA-I in HDL

**Figure 2:**
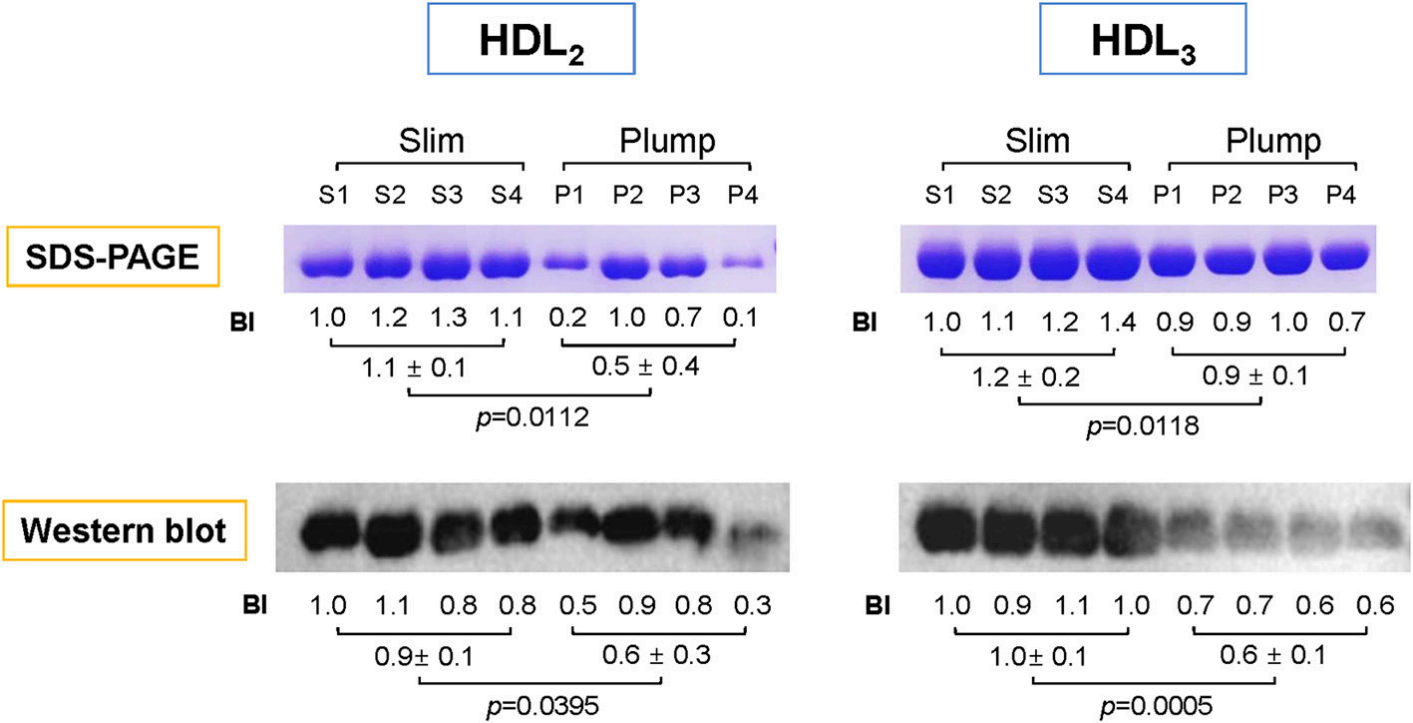
Electrophoretic patterns of apolipoproteins in HDL_2_ and HDL_3_. The number indicates band intensity (BI) of apoA-I from densitometric analysis using Gel-Doc (Bio-Rad). The expression level of apoA-I was compared by Western blotting. BI was calculated using Chemi-Doc (Bio-Rad).

To compare the expression level of apoA-I in HDL, the same amount of total proteins per individual HDL was loaded on each lane of a gel. As shown in Figure 2, the slim group showed 2.2- and 1.3-fold higher expression levels of apoA-I in HDL_2_ and HDL_3_, respectively, compared to the plump group as determined by SDS-PAGE. Based on Western blotting with an apoA-I antibody, the slim group showed 1.5- and 1.6-fold higher apoA-I levels in HDL_2_ and HDL_3_, respectively, compared to the plump group.

### HDL_2_ particle size and cholesterol efflux

**Figure 3:**
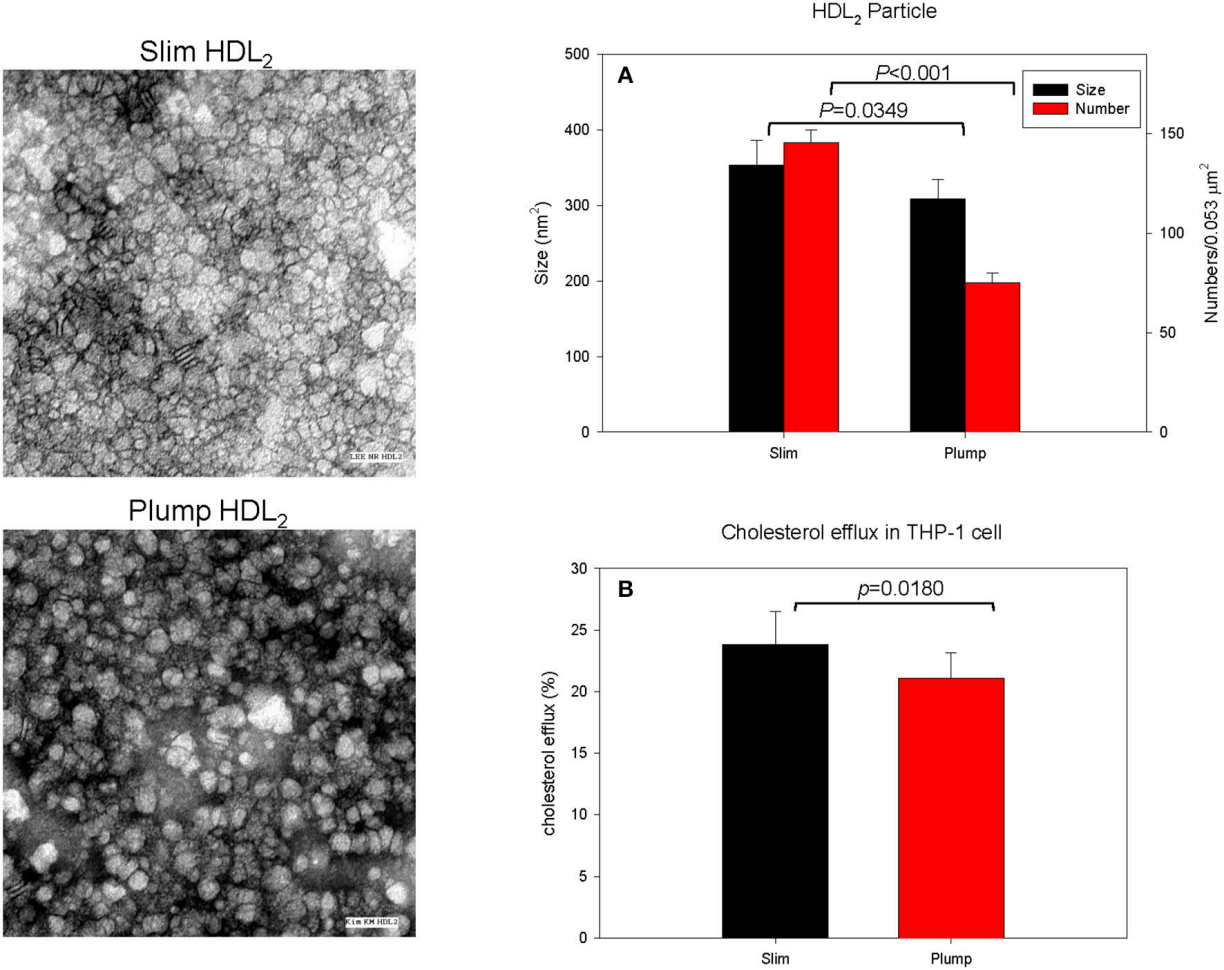
Cholesterol efflux activity and a representative photo of negatively-stained HDL_2_ and HDL_3_ from slim and plump groups (electron microscopy). All micrographs are shown at a magnification of 40,000×. The scale bar corresponds to 100 nm. **(A)** Shows measured particle size of HDL and particle number in the designated area. **(B)** Shows cholesterol efflux activity from macrophages.

The slim group showed a 15% larger HDL particle size and 1.9-fold higher number of particles than the plump group as shown in Figure 3A and a representative photo of Figure 3 from TEM analysis. HDL_2_ from the slim group showed significantly enhanced cholesterol efflux activity from macrophages (around 24 ± 2% efflux), whereas the plump group showed 21 ± 1% efflux (*p* = 0.018) during 24 h of incubation of each HDL as shown in Figure 3B.

### Antioxidant ability

**Figure 4:**
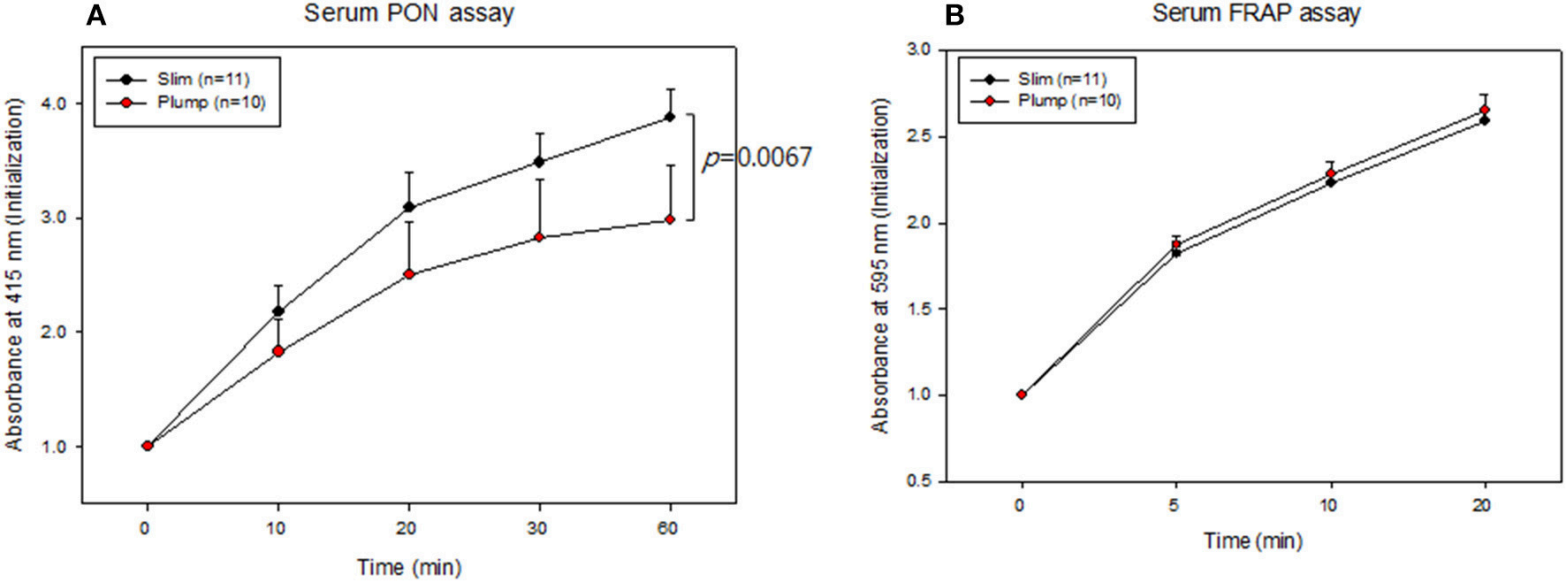
Serum antioxidant activity. Paraoxonase activity **(A)** and ferric ion reduction ability **(B)**.

Serum PON activity of the slim group was 1.3-fold higher than that of the plump group up to 60 min of incubation, as shown in Figure 4A. Serum FRAP ability was not significantly different between the groups, and the two groups showed saturated reduction potential at the same incubation time, as shown in Figure 4B. The slim group showed higher HDL-associated antioxidant enzyme activity, although their antioxidant potentials in serum were similar. This result suggests that HDL from the slim group showed enhanced functionality.

**Figure 5:**
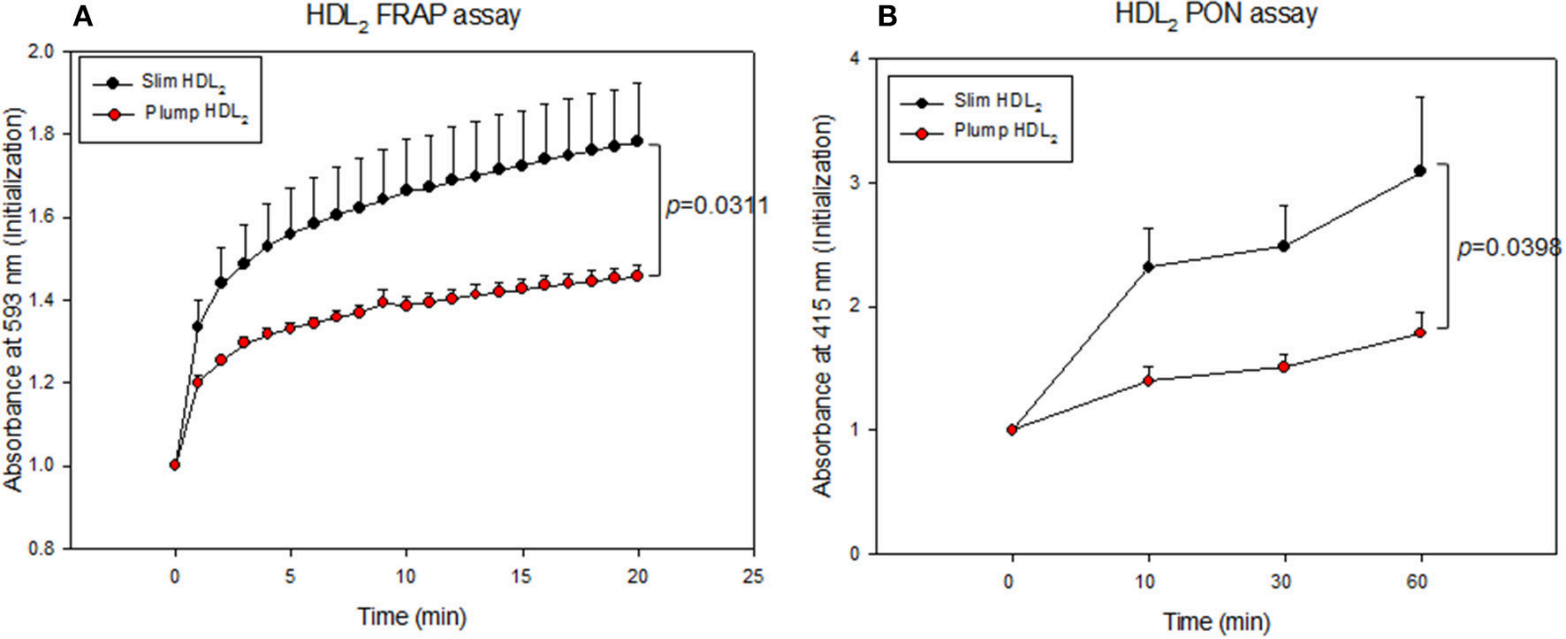
Antioxidant activity of HDL_2_-associated enzymes. Ferric ion reduction ability **(A)** and Paraoxonase activity **(B)**.

The slim group also showed higher HDL-associated antioxidant activity than the plump group (Figure 5). Regarding FRAP ability, the slim group showed 22% higher reduction potential than the plump group during 20 min of incubation (Figure 5A). Additionally, the slim group showed 1.7-fold higher PON activity than the plump group during 60 min of incubation (Figure 5B).

### Compositions of lipoproteins

**Figure 6:**
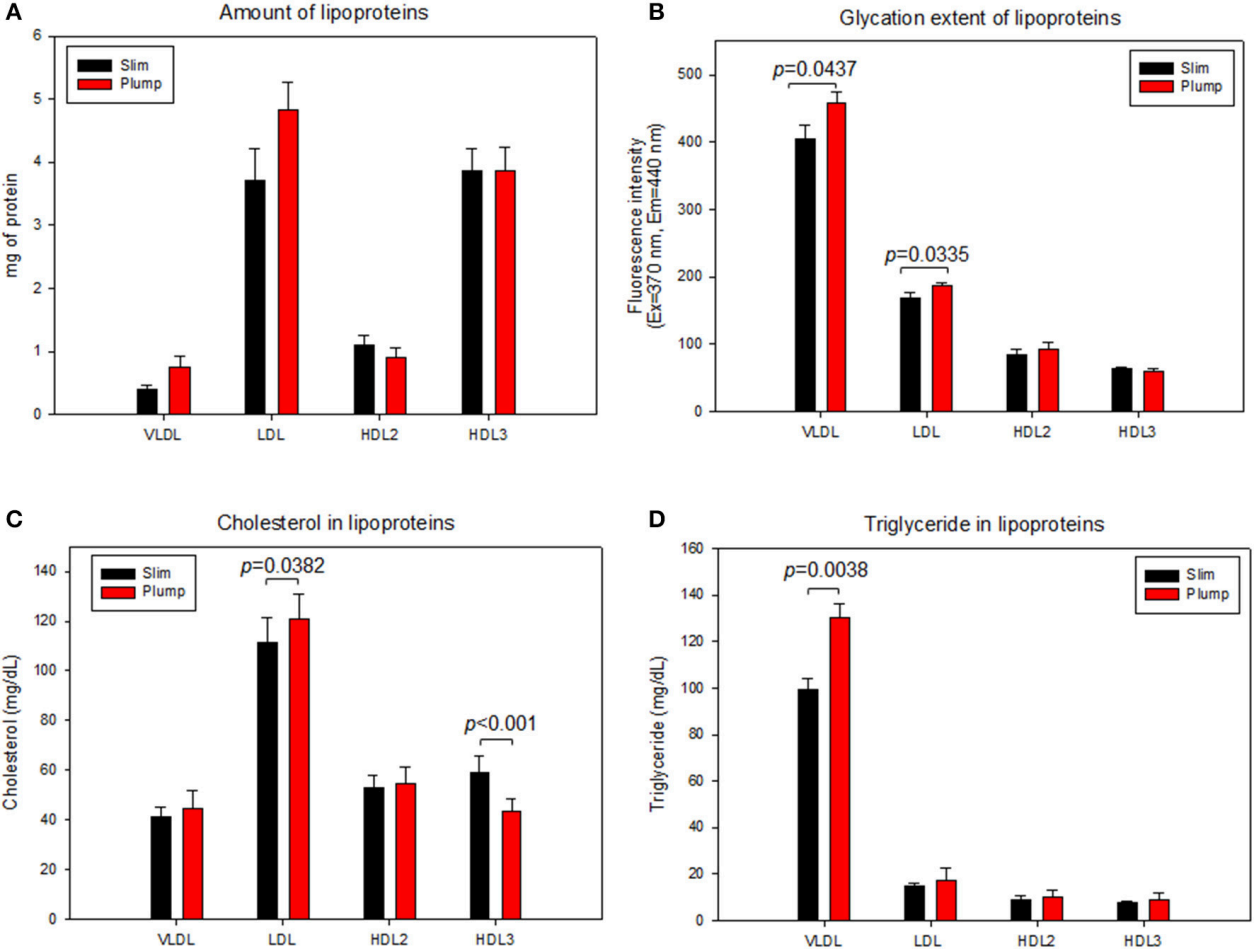
Lipoprotein composition and glycation extent of slim and plump groups. Protein content **(A)** and glycation extent **(B)**. Lipoprotein composition and glycation extent of slim and plump groups. Cholesterol **(C)** and triglyceride content **(D)**.

As shown in Figure 6A, the plump group showed 1.9- and 1.3-fold higher protein contents in VLDL and LDL, respectively, but 20% lower protein content in the HDL_2_ fraction. The lower protein content in HDL_2_ from the plump group was strongly associated with reduced serum apoA-I content (Table 2). However, the plump group showed 10–13% higher glycation extent in all lipoproteins, especially VLDL and LDL (Figure 6B). At the same protein amount, the plump group showed 9% higher cholesterol content in VLDL and LDL (Figure 6C). However, the plump group showed 27% less cholesterol content in HDL_3_, whereas there was no difference in HDL_2_-C. Interestingly, the plump group showed elevated TG contents in all lipoprotein fractions, especially 1.3- and 1.2-fold higher TG contents in VLDL and HDL_3_, respectively. HDL_3_ from the plump group showed higher TG and lower cholesterol contents than the slim group (Figure 6D), suggesting functionality of lipoproteins may be impaired at the beginning of overweight status.

### Modification of lipoproteins

**Figure 7:**
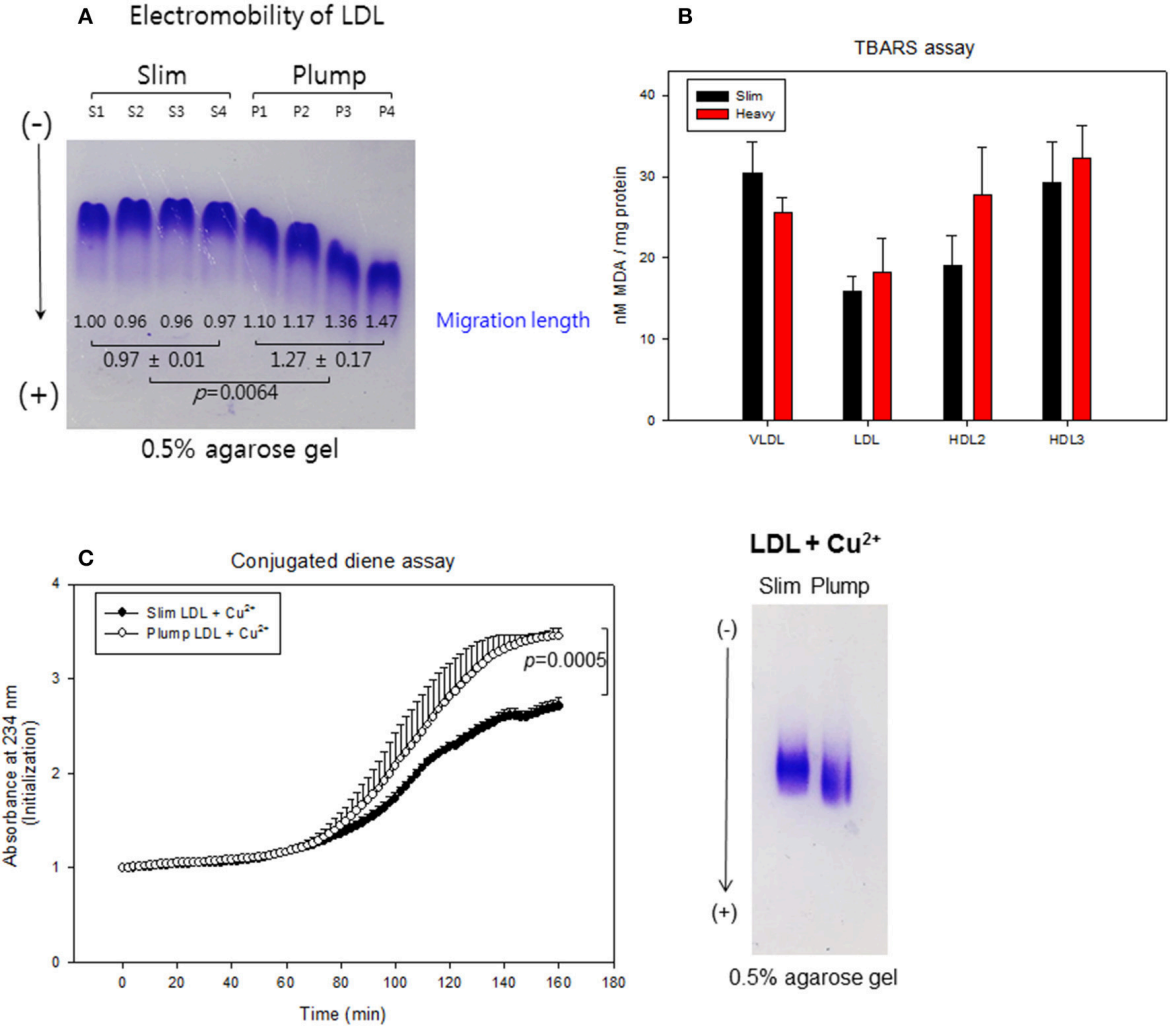
Lipoprotein properties between slim and plump groups. Electromobility of LDL **(A)**, extent of oxidized species **(B)**. Susceptibility to cupric ion mediated oxidation **(C)**.

LDL from the plump group showed faster electromobility, as shown in Figure 7A, due to putative modifications such as oxidation and glycation. The plump group showed greater migration distance (1.3 ± 0.2 cm) than the slim group (0.9 ± 0.1 cm). TBARS assay was performed to determine oxidized products, showed all lipoproteins from the plump group showed higher MDA contents (Figure 7B). Especially, the plump group showed 45% more MDA content in HDL_2_ compared to the slim group.

Cupric ion treatment revealed that the plump group was 1.3-fold more sensitive to oxidation than the slim group (Figure 7C) during 150 min of incubation. After incubation, Cu^2+^-treated LDL from the plump group showed faster electromobility with fainter band intensity as shown in the photo of Figure 7C, suggesting that LDL from the plump group was more susceptible to oxidation.

### Uptake of LDL into macrophages

After 48 h of treatment with each LDL, the plump group showed 2-fold higher oil-red O-stained area than the slim group (Supplementary Figure 4), suggesting that LDL from the plump group was taken up 2-fold more into macrophages. This result supports the earlier findings that LDL from the plump group was more glycated (Figure 6B) and oxidized (Figure 7), as modified LDL was more phagocytosed by macrophages to accelerate foam cell production.

### Embryo survivability

Microinjection of LDL from the plump group caused the most severe embryo death up to 69% survival, whereas LDL from the slim group resulted in 75% embryo survival at 48 h post-injection, as shown in Supplementary Figure 5B. This difference in embryo survival can be attributed to more oxidized in LDL from the plump group (Figure 7). DHE staining revealed that plump LDL-injected embryos showed the strongest red intensity, suggesting the highest production of reactive oxygen species (Supplementary Figures 5A,C).

Injection of oxLDL caused the lowest embryo survival (around 44%), whereas PBS injection resulted in 84% survival, as shown in the graph of Supplementary Figure 6. In the presence of oxLDL, co-injection of HDL_3_ from the slim group enhanced survival (around 70%), whereas HDL_3_ from the plump group resulted in lower survivability (around 59%). oxLDL alone-injected embryos showed the strongest red fluorescence, which was 3-fold higher than PBS-injected embryos. However, HDL_3_ from the slim and plump groups caused 25 and 16% reduction of red fluorescence, respectively, suggesting that HDL from the slim group was more effective in preventing embryonic death and ROS production mediated by oxLDL. Taken together, the plump group showed more dysfunctional HDL with smaller particle size, reduced antioxidant ability, greater atherogenic properties, such as higher CETP activity and mass with more inflammatory properties in human cells and zebrafish embryos.

## Discussion

To our knowledge, this is the first report demonstrating differences in lipoproteins and lipid levels between young women with slim and plump body weights. It has been well established that overweight and obese adolescents are worldwide epidemic issue (34) due to the prevalence of hypertension, cardiovascular disease, and metabolic syndrome, etc. However, there are few drugs capable of treating obesity long-term other than orlistat (Xenical) and lorcaserin (Belviq), which have several side effects such as physical and emotional disorders (35).

Elevated BP is the most frequent comorbidity identified in overweight adolescents (36). Our current results clearly show that % HDL-C was inversely correlated with body weight, body fat mass, and blood pressure (Supplementary Figures 1–3). However, there were no correlations with the amount of HDL-C (mg/dL), suggesting that % HDL-C in TC is a reliable biomarker to predict body fat mass and blood pressure (Table 2). There is little information about lipoprotein profiles and obesity in young women. Furthermore, there has been no report about the lipid and lipoprotein profiles of young women with a moderately plump body weight. Until now, there have been limitations in interpreting the physiological roles of HDL in health and beauty since most studies have focused on the quantity of HDL-C in order to explain its beneficial activities.

Recently, the importance of HDL functionality has been well investigated to prevent CVD and metabolic syndrome via protection of LDL from modification (37). In our study, the slim group showed enhanced antioxidant activity in both serum and HDL (Figures 4, 5) with less oxidation of LDL (Figure 7). Although the plump group showed lower protein content in HDL_2_, its proteins were more glycated and modified (Figure 6). Further, although the two groups showed a difference of 8.4 ± 2 kg in body weight and the plump group had a normal BMI, their lipid profiles and lipoprotein properties were distinctly different in terms of CETP mass and HDL functionality.

Elevated levels of CETP activity was found to be a major determinant of the atherogenic dyslipidemia, and atherosclerotic cardiovascular disease (38). Our study have also reported an elevated level of CETP mass and %CE-transfer in the plump group which was found to be significant when compared with the slim group. CETP is that enzyme accountable for moving cholesterol esters and triglycerides between, LDL, VLDL, and HDL. Lower level of CETP nurture HDL formation. Considering higher HDL levels are associated with lower risk of atherosclerosis, the elevated level of CETP is thought to play in promoting the disease by lowering in HDL-C (39). CETP mass activity was directly correlated with body weight and VFM (Supplementary Figure 2B), whereas apoA-I mass was inversely correlated with VFM (Tables 1, 2). In a Japanese study, obese children showed increased plasma CETP activity as well as lower apoA-I content and higher serum TG levels (40). Another study on obese Japanese adults (BMI 33.1 kg/m^2^) reported increased serum TG and reduced HDL-C levels via elevation of CETP activity (12). Further, obese women with BMIs around 35.1 ± 0.7 kg/m^2^ showed 1.22-fold higher CETP content in serum compared to lean women (22.4 ± 0.3 kg/m^2^) along with 1.7-fold higher serum TG and 23% lower HDL-C levels (41). These reports are in good agreement with our current results in terms of lowered HDL-C and increased TG serum levels via elevation of CETP mass, although subjects in the current study were not obese. The higher percentage of HDL-C in the slim group was associated with lower body fat and VFM contents. In both groups, the percentages of HDL-C and apoA-I contents were inversely correlated with blood pressure. VLDL and LDL from the plump group showed 13 and 30% more glycated end products and TG.

Injection of LDL from the slim group into zebrafish embryos resulted in 75% survival, whereas the overweight group showed 70% survival, indicating that LDL from the plump group had more inflammatory properties. The lowest embryo survivability (around 44%) was seen after injecting oxLDL while PBS injection lead to 84% survivability. The co-injection of HDL_3_ and oxLDL in the slim group enhanced the survivability up to 70% in the zebrafish embryos, although the oxLDL and HDL_3_ from the plump group resulted a lower survivability of 59%. The study observed that the HDL_3_ from the slim and plump groups elicited 25 and 16% reduction in the red fluorescence that suggested a protective role of HDL extracted from slim group in the embryonic death and ROS production.

Further, the slim group showed 22% higher antioxidant activity in serum and HDL as well as 26% higher apoA-I content in HDL than the plump group. ApoA-I content in HDL from the slim group was 2-fold higher while LDL from the slim group was less oxidized compared to the plump group. LDL from the plump group showed 2-fold greater uptake into macrophages, whereas HDL from the plump group did not prevent ox-LDL phagocytosis by macrophages.

Although obese patients are often associated with low serum HDL-C and antioxidant activities, there has been no information about HDL particle number and size. The current results show that changes in obese status were associated with smaller HDL particle size and number as well as lowered apoA-I content. In addition to HDL, LDL showed a smaller particle size with increased susceptibility to oxidation. It is well known that PON-1 hydrolyzes lipid peroxides in order to prevent oxidation of LDL. Reduced HDL-associated PON-1 activity in the plump group (Figure 5) can explain the increase in LDL oxidation (Figure 7) and phagocytosis by macrophages (Supplementary Figure 4). Similar to our current results, obese patients displaying obstructive sleep apnea showed reduced serum PON-1 activities with elevated oxLDL levels, inflammation, and endothelia dysfunction (42). Impairment of antioxidant ability is well correlated with reduction of apoA-I content in HDL (43). It has been reported that obesity and metabolic syndrome are frequently associated with reduced apoA-I and elevated apoC-III contents in HDL along with decreased particle size (44). Plasma apoA-I level has been shown to be inversely associated with obesity in the Framingham Offspring Study conducted on 4,260 young adult men and women (45). Although apoA-I level was lower in the plump group in the current study, there was no difference in apoC-III level since the plump group was not obese.

In older men, diet-induced weight loss has been shown to be associated with increased HDL-C and apoA-I levels as well as reduced body fat mass and CETP activity. Among body fat mass areas, intra-abdominal fat (IAF) and abdominal subcutaneous fat (SQF) were shown to be inversely correlated with apoA-I content, suggesting that body fat mass and distribution are intimately associated with HDL functionality (46). Furthermore, older men showed an increased LDL particle size, whereas HDL particle size was not reported.

Obese subjects show smaller LDL and HDL particle sizes (47). In a Turkish study, obese children showed lower serum HDL-C and higher TG levels than the control despite similar TC. They also showed lower PON activity, increased BMIs, and reduced HDL-C content (48), which are in good agreement with the current results. To prevent obesity progression, HDL should be functionally enhanced as well as increased in particle size via reduction of CETP activity. A cohort study studying the hearts of young overweight subjects (2,017 participants, aged 12–15-years-old) reported higher serum C-reactive protein, serum amyloid A, and CETP activities. Interestingly, the overweight group showed 1.3-fold higher HDL_2_-associated CETP activity, whereas there was no difference in HDL_3_-associated CETP activity (49), similar to our current results.

## Conclusions

In conclusion, despite having normal BMIs, the plump group showed more atherogenic features in LDL and HDL as well as elevated oxidation and glycation with loss of antioxidant ability and apoA-I. Elevation in the CETP might be a key player in HDL metabolism during obesity progression, accumulating in visceral fat to impair the lipoprotein levels, antioxidant activity, and lipoprotein functionality. It is necessary to monitor the quality of HDL in terms of composition, structure and functional properties in order to increase the particle size and antioxidant activity.

## Ethics statement

Informed consent was obtained from all participants prior to enrollment in the study, and the Institutional Review Board at Yeungnam University (Gyeongsan, South Korea) approved the protocol (IRB #7002016-A-2015-003).

## Author contributions

K-HP, DY, S-JK performed the experiments. J-RK analyzed the data. K-HC wrote the manuscript and supervised the whole project.

### Conflict of interest statement

The authors declare that the research was conducted in the absence of any commercial or financial relationships that could be construed as a potential conflict of interest.
