# Supplementary figures and images for: Slim Body Weight Is Highly Associated With Enhanced Lipoprotein Functionality, Higher HDL-C, and Large HDL Particle Size in Young Women
Source: Front Endocrinol (Lausanne). 2018 Jul 19;9:406. doi: 10.3389/fendo.2018.00406 (PMC6060307; doi:10.3389/fendo.2018.00406)

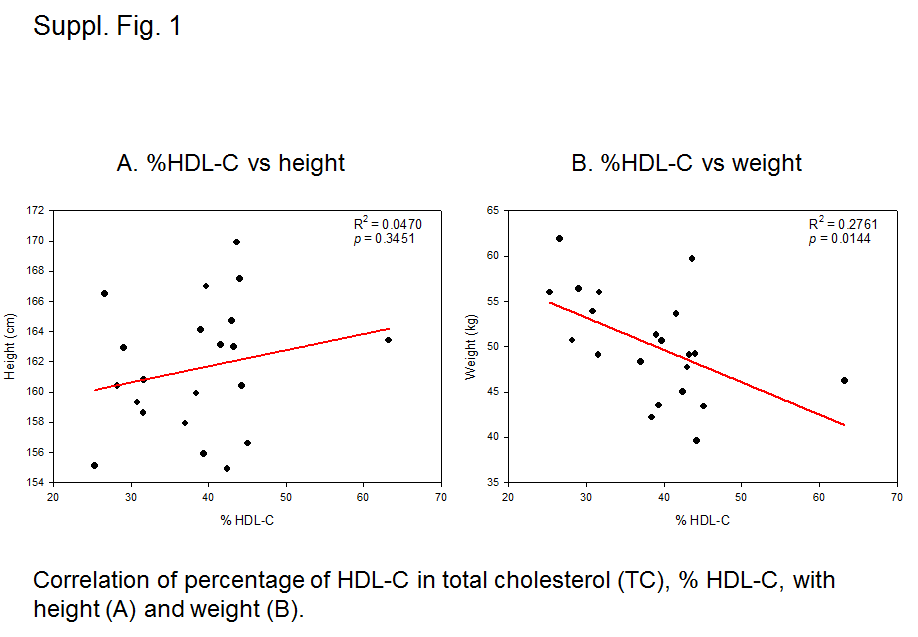

Supplement: Supplementary file 1 [file Image_1.TIF]

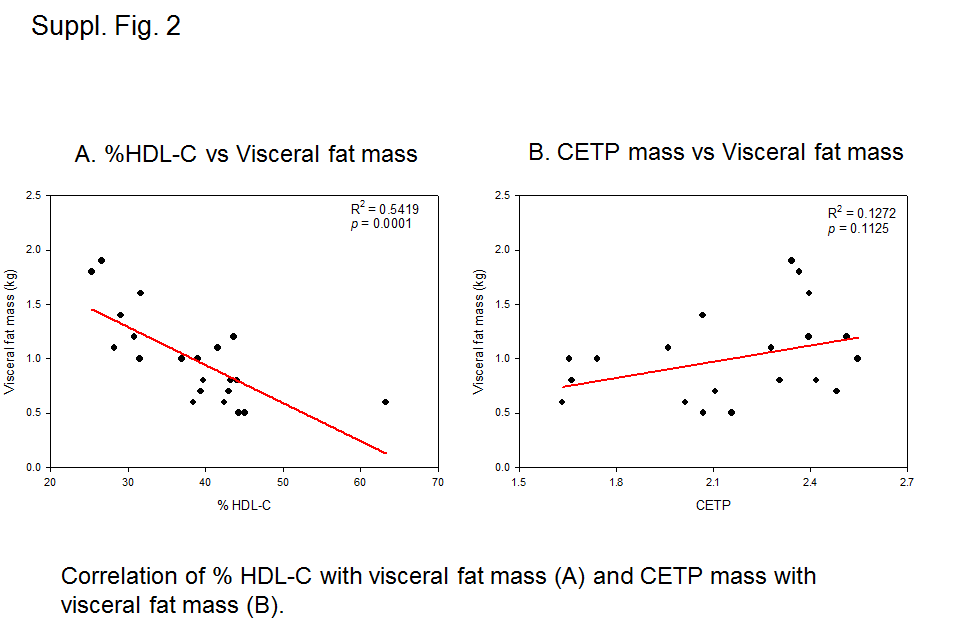

Supplement: Supplementary file 2 [file Image_2.TIF]

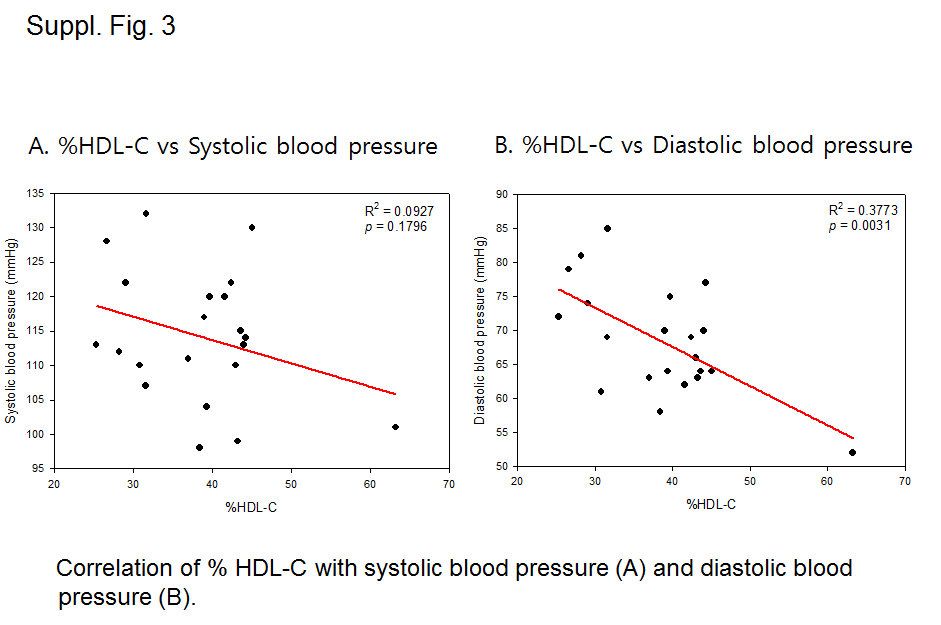

Supplement: Supplementary file 3 [file Image_3.TIF]

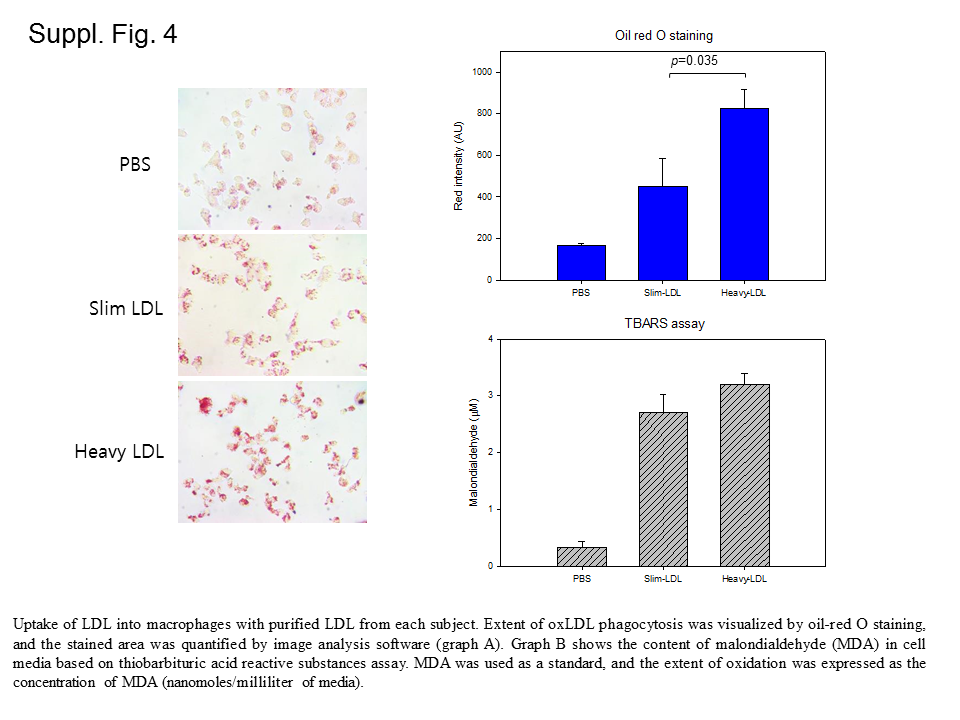

Supplement: Supplementary file 4 [file Image_4.TIF]

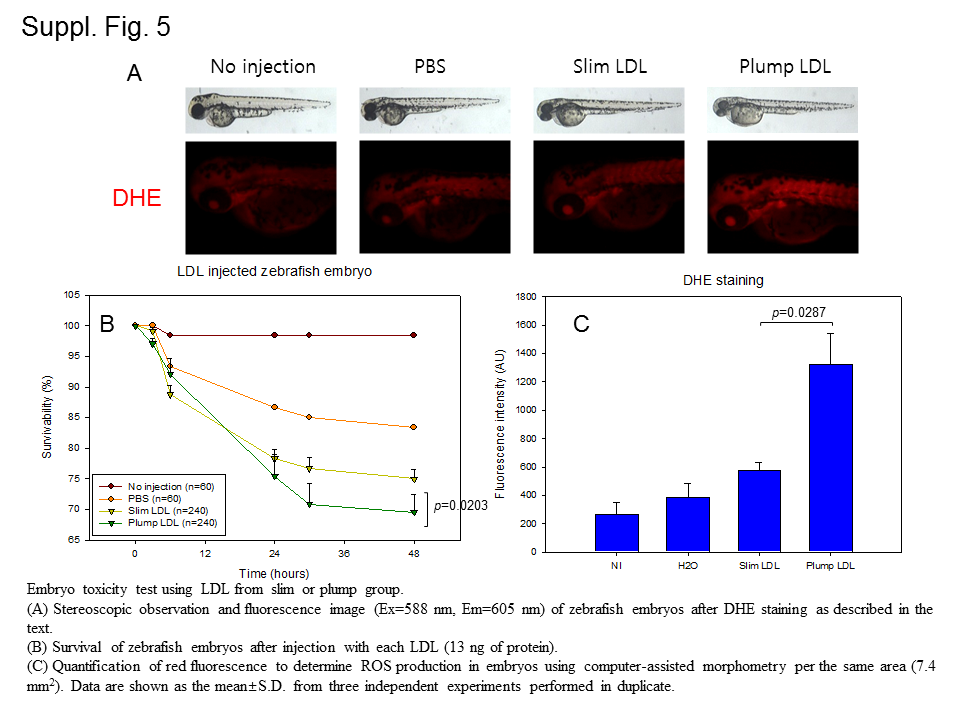

Supplement: Supplementary file 5 [file Image_5.TIF]

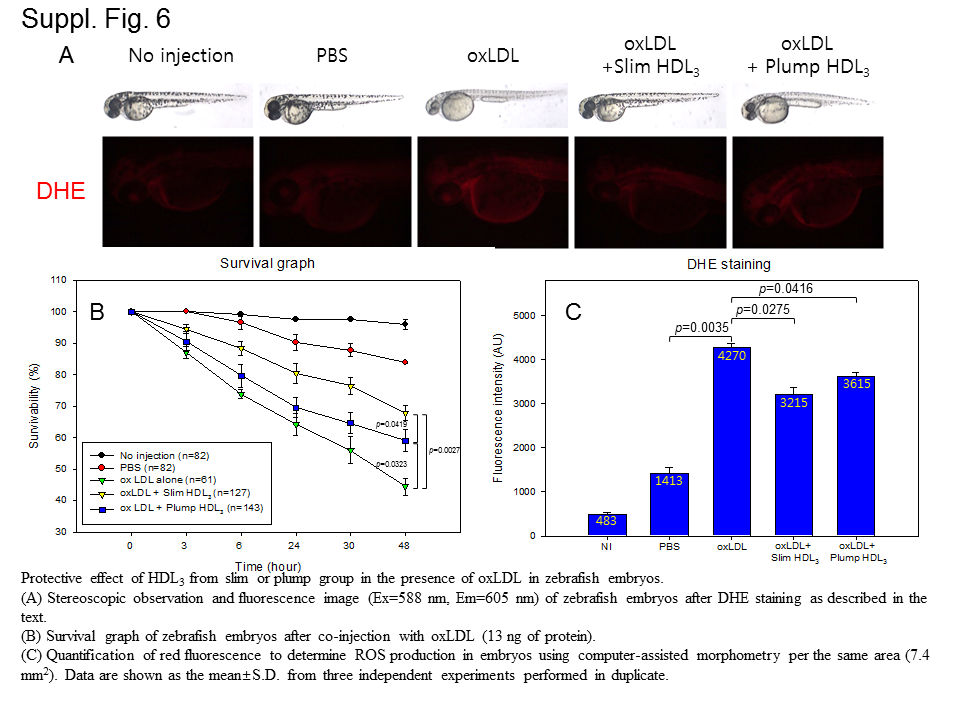

Supplement: Supplementary file 6 [file Image_6.TIF]
